# 5,2′-Dibromo-2,4′,5′-trihydroxydiphenylmethanone Inhibits LPS-Induced Vascular Inflammation by Targeting the Cav1 Protein

**DOI:** 10.3390/molecules27092884

**Published:** 2022-04-30

**Authors:** Hongxia Yuan, Qianyi Hou, Xiue Feng, Yuanlin Zhang, Fan Yang, Rui Ge, Qingshan Li

**Affiliations:** 1 Shanxi Key Laboratory of Innovative Drug for the Treatment of Serious Diseases Basing on the Chronic Inflammation, Shanxi University of Chinese Medicine, Jinzhong 030619, China; yuanhongxia609@sxtcm.edu.cn (H.Y.); houqianyi@sxtcm.edu.cn (Q.H.); zhangyl@sxtcm.edu.cn (Y.Z.); 2 School of Pharmaceutical Science, Shanxi Medical University, Taiyuan 030001, China; xiuefeng@sxmu.edu.cn (X.F.); fanfan42@sxmu.edu.cn (F.Y.); cpugr@126.com (R.G.)

**Keywords:** 5,2′-dibromo-2,4′,5′-trihydroxydiphenylmethanone, lipopolysaccharide, vascular inflammation, caveolin-1, EA.hy926 cells, NF-κB signaling

## Abstract

Vascular inflammation is directly responsible for atherosclerosis. 5,2′-Dibromo-2,4′,5′-trihydroxydiphenylmethanone (TDD), a synthetic bromophenol derivative, exhibits anti-atherosclerosis and anti-inflammatory effects. However, the underlying pathways are not yet clear. In this study, we first examined the effects of TDD on toll-like receptor-4 (TLR4) activity, the signaling receptor for lipopolysaccharide (LPS), and found that TDD does not inhibit LPS-induced TLR4 expression in EA.hy926 cells and the vascular wall in vivo. Next, we investigated the global protein alterations and the mechanisms underlying the action of TDD in LPS-treated EA.hy926 cells using an isobaric tag for the relative and absolute quantification technique. Western blot analysis revealed that TDD inhibited NF-κB activation by regulating the phosphorylation and subsequent degradation IκBα. Among the differentially expressed proteins, TDD concentration-dependently inhibited Caveolin 1(Cav1) expression. The interaction between Cav1 and TDD was determined by using biolayer interference assay, UV-*vis* absorption spectra, fluorescence spectrum, and molecular docking. We found that TDD can directly bind to Cav1 through hydrogen bonds and van der Waals forces. In conclusion, our results showed that TDD inhibited LPS-induced vascular inflammation and the NF-κB signaling pathway by specifically targeting the Cav1 protein. TDD may be a novel anti-inflammatory compound, especially for the treatment of atherosclerosis.

## 1. Introduction

In recent years, cardiovascular disease (CVD) is still the leading reason for human death worldwide with the increase of its incidence and mortality, accounting for a huge financial and social toll [1]. The main cause of CVD is atherosclerosis, which is a chronic inflammatory vascular disorder [2]. Inflammation plays a critical role in the development and progression of atherosclerosis [3,4,5]. Thus, inhibiting the inflammatory response may be a potential therapeutic approach for treating atherosclerosis. Among the mechanisms involved, the endotoxin lipopolysaccharide (LPS) activates several inflammatory pathways that accelerate the formation of atheromatous lesions in animals and humans [6,7,8,9,10,11,12]. LPS is an important virulence factor of gram-negative bacteria that produces inflammation and endothelial injury [13,14]. Therefore, inhibiting the inflammatory pathways activated by LPS may be a potential therapeutic approach for treating atherosclerosis [15,16].

Caveolin 1(Cav1) is an integral membrane protein involved in endocytosis, cholesterol homeostasis, signal transduction, and mechanoprotection [17,18]. Previous studies revealed a critical role of Cav1 in the progression of atherosclerosis [19,20,21,22]. Cav1 knockout mice had decreases in atherosclerosis [21,23,24,25], while re-expression Cav1 in the endothelium promoted atherosclerotic lesions [26,27]. An increased Cav1 expression has been observed in atherosclerotic lesions of hypercholesterolemic animals and in endothelial cells from smokers [28,29,30]. The upregulation of Cav1 gene expression favored vascular inflammation and cell apoptosis, thereby contributing to the occurrence of atherosclerosis [20].

5,2′-dibromo-2,4′,5′-trihydroxydiphenylmethanone (TDD), a marine bromophenol derivative, exerts anti-atherogenic effects, anti-oxidative, anti-apoptotic, and anti-inflammatory [31,32,33,34]. Although recent studies investigated the mechanisms by which TDD attenuates LPS-induced inflammation [32], the molecular pathways implicated remain to be defined.

In this study, we aimed at investigating the biological effects of TDD during an LPS-induced inflammatory response in EA.hy926 cells and the vascular wall.

## 2. Results

### 2.1. Effects of TDD on the Expression of TLR4 in LPS-Treated EA.hy926 Cells

**Figure 1:**
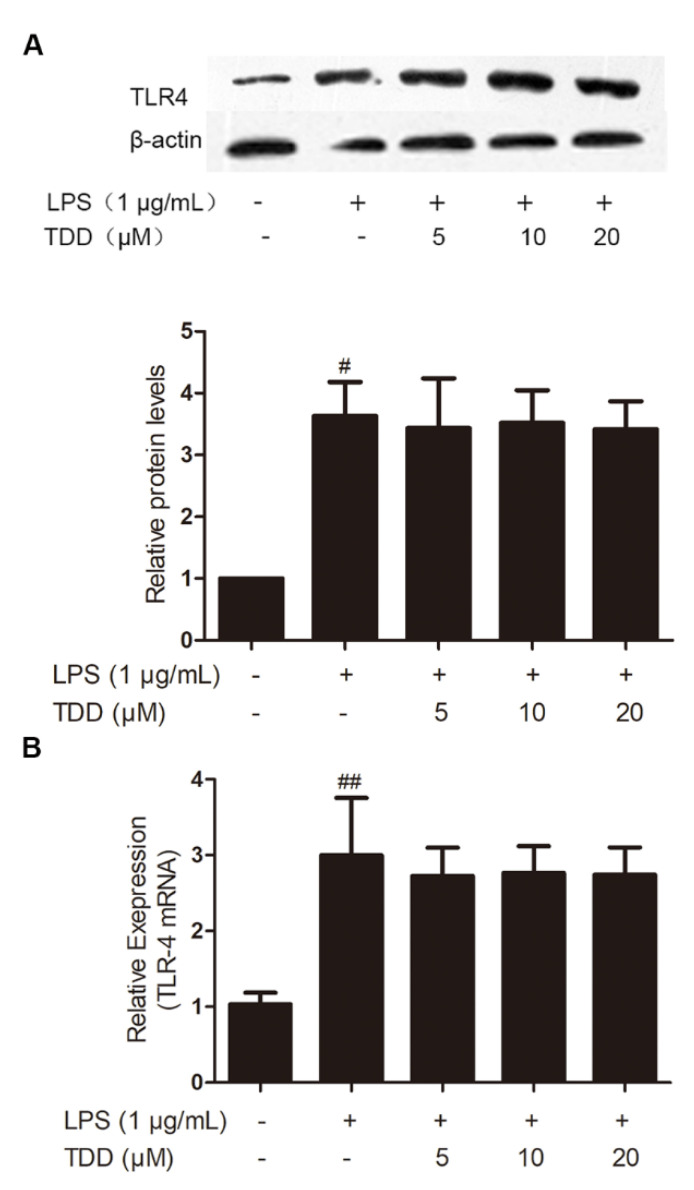
Effects of TDD on the expression of TLR4 in LPS-stimulated EA.hy926 cells. EA.hy926 cells were treated with various concentrations of TDD (5, 10, and 20 µM) for 4 h, and then LPS (1 μg/mL) was added for 12 h. (**A**) protein levels, and (**B**) mRNA levels. The data show the mean ± S.D. of three independent experiments. ^#^ *p* < 0.05 and ^##^ *p* < 0.01, compared to the LPS (−) TDD (−) group.

As shown in Figure 1, LPS exposure significantly increased on the expression levels of TLR4 mRNA and proteins, whereas TDD did not inhibit LPS-induced TLR4 expression.

### 2.2. Effects of TDD on the Expression of TLR4 in the Aortae of LPS-Treated Mice

**Figure 2:**
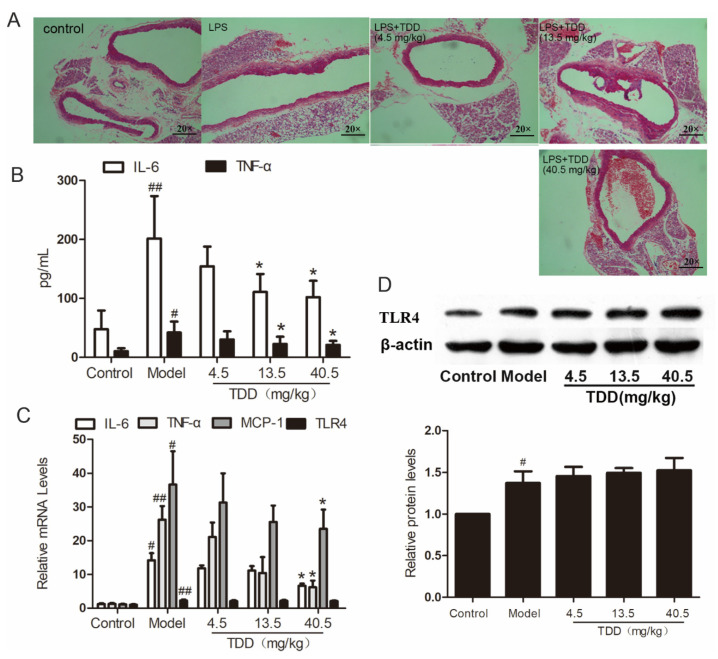
TDD attenuates LPS-induced vascular inflammation in the aortae of BALB/c mice. (**A**) Changes of aorta histomorphology of mouse (staining, 20 × 10), (**B**) effects of TDD on the serum IL-6 and TNF-α, (**C**) effects of TDD on the serum TNF-α, IL-6, and MCP-1 in aorta, and (**D**) effects of TDD on TLR4 protein levels and mRNA levels. Data are presented as mean ± SD (*n* = 6). * *p <* 0.05 compared with the Model group. ^#^ *p* < 0.05 and ^##^ *p* < 0.01 compared with the Control group.

As shown in Figure 2A, the vascular tissues of LPS-treated mice exhibited obvious inflammatory cell infiltration. Interestingly, TDD attenuated LPS-induced inflammatory cell infiltration in a dose-dependent manner.

Further, the serum concentrations of the typical inflammatory cytokines tumor necrosis factor alpha (TNFα) and interleukin- 6 (IL-6) were analyzed using ELISA. As shown in Figure 2B, pretreatment with TDD decreased the secretion of IL-6 and TNF-α in serum obtained from LPS-treated mice. Meanwhile, the mRNA levels of TNF-α, IL-6, Monocyte chemotactic protein 1(MCP-1), and TLR4 were increased in the aortae of LPS-treated mice and attenuated by pretreatment with TDD; however, the mRNA levels of TLR4 remained unchanged by this treatment (Figure 2C). The inhibitory effect of TDD on LPS-induced TLR4 expression was consistent with the mRNA levels; this was further confirmed by Western blotting (Figure 2D).

### 2.3. Effects of TDD on Protein Expression

**Figure 3:**
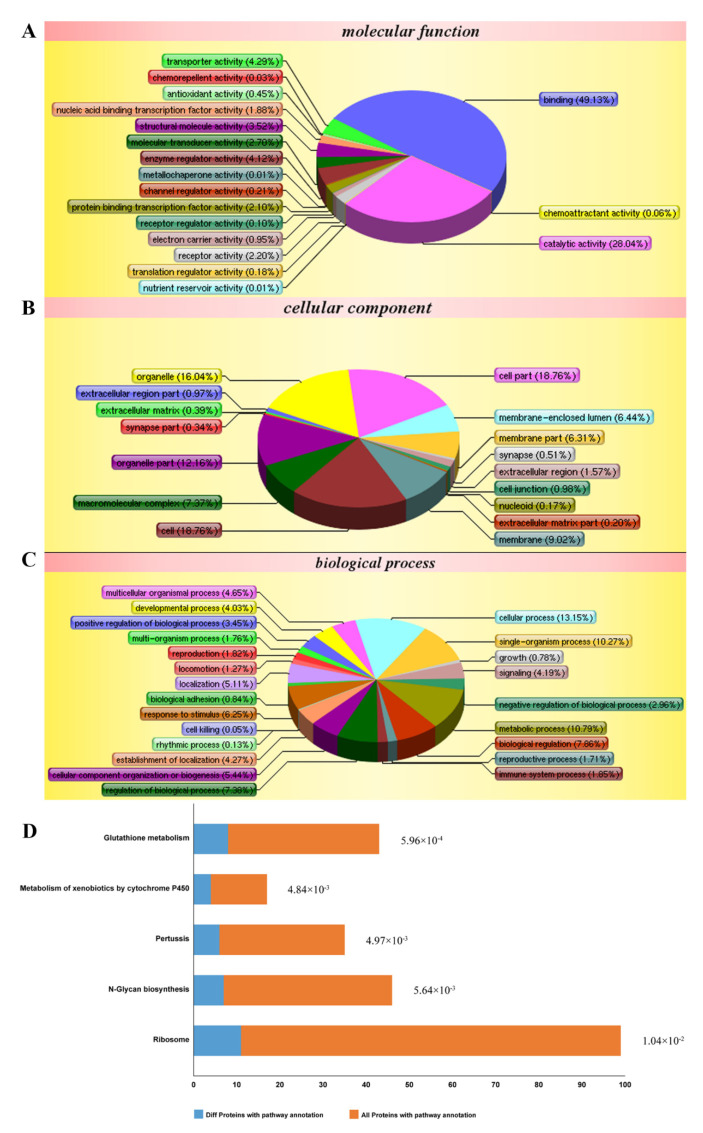
Differentially expressed proteins impacted by LPS and TDD treatment compared to LPS treatment in EA.hy926 cells were analyzed according to gene ontology in three dimensions and KEGG pathways: (**A**) biological processes, (**B**) cellular localization, (**C**) the molecular functions, and (**D**) KEGG pathways. EA.hy926 cells were treated with TDD (10 µM) for 4 h, and then LPS (1 μg/mL) was added for 12 h.

According to the isobaric tags for relative and absolute quantification (iTRAQ)-based proteomic analysis, a total of 5155 proteins were identified. Among them, 288 differentially expressed proteins were obtained by comparing between cells treated with LPS and TDD and cells treated only with LPS. We categorized the 288 proteins into three functional groups: molecular functions, cellular components, and biological processes. As shown in Figure 3A–C, the molecular functions were binding processes, catalytic activities, transporter activities, enzyme regulator activities, and so on. The cellular components were the cell, the cellular membrane, organelles, macromolecular complexes, and so on. The biological processes were cellular processes, single-organism processes, regulation processes, responses to stimuli, and so on. The most relevant pathways of differentially expressed proteins were glutathione metabolism, metabolism of xenobiotics by cytochrome P450, pertussis toxin-related pathway, N-glycan biosynthesis pathway, and ribosome pathway (Figure 3D). Furthermore, the pertussis toxin-related pathway was related to NF-κB signaling (Supplementary Figure S1).

### 2.4. Validation of the Activity of the NF-κB Signaling

**Figure 4:**
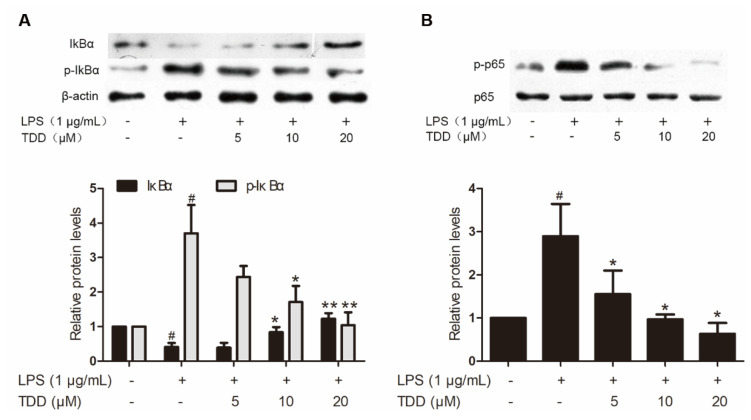
Effects of TDD on LPS-induced NF-κB activation in EA.hy926 cells. EA.hy926 cells were treated with various concentrations of TDD (5, 10, and 20 µM) for 4 h, and then LPS (1 μg/mL) was added for 0.5 h. (**A**) Effects of TDD on IκBα activation and IκBα degradation in LPS-stimulated EA.hy926 cells, and (**B**) effects of TDD on p65 activation in LPS-stimulated EA.hy926 cells. The data show the mean ± S.D. of three independent experiments. * *p* < 0.05 and ** *p* < 0.01, compared to the LPS (+) TDD (−) group. ^#^ *p* < 0.05 compared to the LPS (−) TDD (−) group.

Western blotting found that TDD reduced LPS-induced increases in p65 and IκBα phosphorylation (Figure 4). In addition, there was a significant increase of LPS-induced IκBα degradation.

### 2.5. Effects of TDD on Selected Proteins 

**Figure 5:**
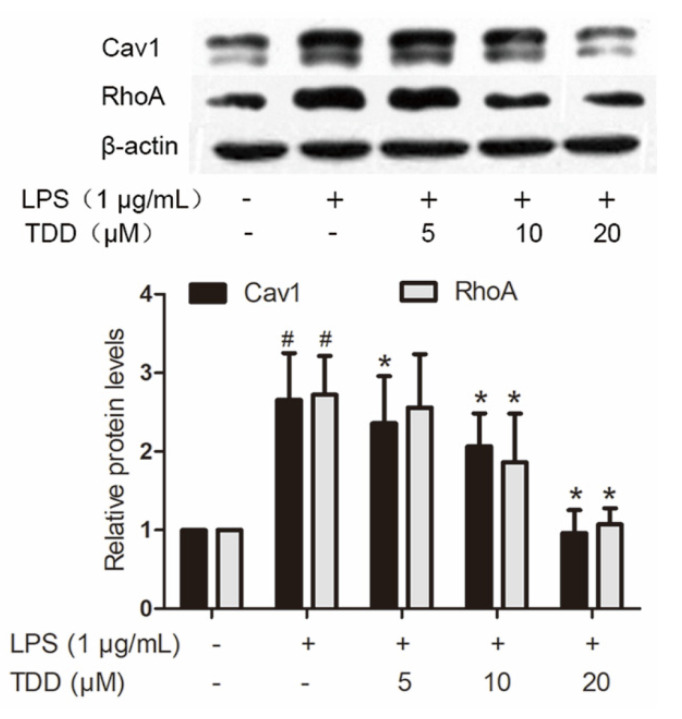
Effects of TDD on the expression of Cav1 and RhoA in LPS-stimulated EA.hy926 cells. EA.hy926 cells were treated with various concentrations of TDD (5, 10, and 20 µM) for 4 h, and then LPS (1 μg/mL) was added for 12 h. The data show the mean ± S.D. of three independent experiments. * *p* < 0.05 compared to the LPS (+) TDD (−) group. ^#^ *p* < 0.05 compared to the LPS (−) TDD (−) group.

LPS caused altered expression of 437 proteins. Of the 63 proteins that exhibited altered expression after TDD treatment, 43 showed a tendency towards restoration to normal levels. Twenty of them were downregulated by TDD, and 23 were upregulated by TDD. Western blotting found that Cav1 and RhoA expression decreased in a concentration-dependent manner in TDD-treated cells (Figure 5).

### 2.6. Effects of TDD on IL-6, IL-1β, MCP-1, and TNF-α Expression in LPS-Treated EA.hy926 Cells with Cav1 Knockdown

**Figure 6:**
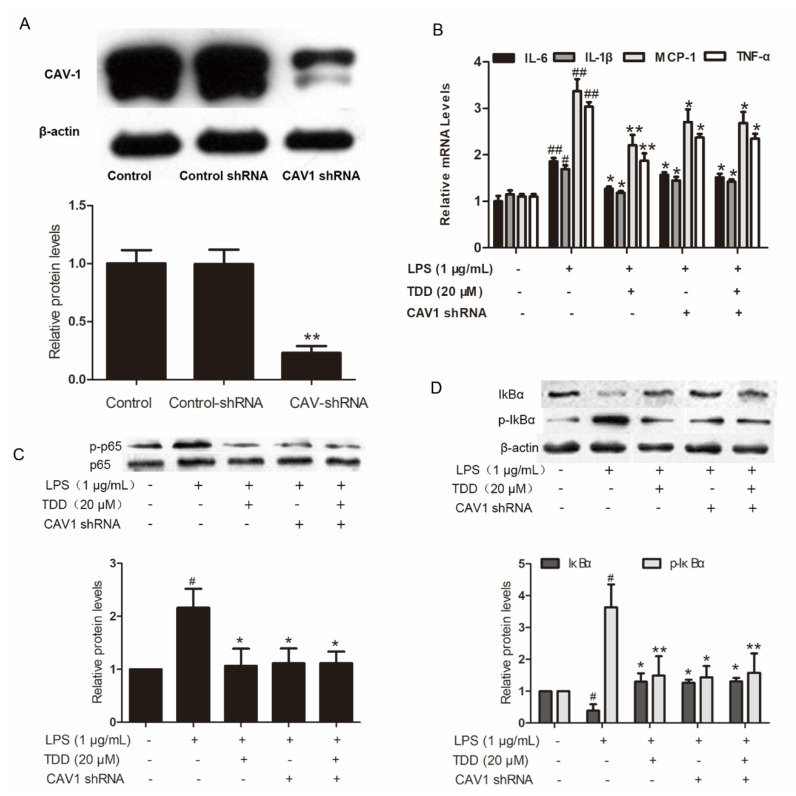
Effects of TDD on the Cav1-NF-κB axis in LPS-treated EA.hy926 cells. (**A**) Effects of shRNA on the expression of Cav1 in EA.hy926 Cells. The data show the mean ± S.D. of three independent experiments. * *p* < 0.05 and ** *p* < 0.01, compared to the control group; (**B**) effects of TDD on LPS-stimulated the IL-6, IL-1β, MCP-1, and TNF-a mRNA expression was determined by real-time RT-PCR with or without transfecting Cav1 shRNA, and; (**C**,**D**) effects of TDD on p65 and IκBα activation and IκBα degradation in LPS-stimulated EA.hy926 cells with or without transfecting Cav1 shRNA. The data show the mean ± S.D. of three independent experiments. * *p* < 0.05, and ** *p* < 0.01, compared to the LPS (+) TDD (−) CAV1 shRNA (−) group. ^#^ *p* < 0.05, ^##^ *p* < 0.01 compared to the LPS (−) TDD (−) group.

The increased levels of IL-6, IL-1β, MCP-1 and TNF-α in LPS-treated EA.hy926 cells transfected with non-targeting control shRNA were significantly attenuated in the TDD group. The levels of IL-6, IL-1β, MCP-1, and TNF-α were also attenuated in LPS-treated EA.hy926 cells transfected with Cav1 shRNA (Figure 6A,B). There was no difference between LPS-treated EA.hy926 cells and TDD + LPS-treated EA.hy926 cells transfected with Cav1 shRNA, suggesting that TDD suppressed IL-6, IL-1β, MCP-1, and TNF-α expression by targeting the Cav1 protein.

### 2.7. Effects of TDD on Inhibitor of NF-κB (IκB) and p65 Phosphorylation in LPS-Treated EA.hy926 Cells with Cav1 Knockdown

IκB and p65 phosphorylation levels, along with IκBα degradation levels, were increased in LPS-treated cells transfected with non-targeting control shRNA. These levels were significantly reduced in the LPS-treated EA.hy926 cells and the TDD + LPS-treated EA.hy926 cells transfected with Cav1 shRNA (Figure 6C,D), indicating that TDD suppressed the LPS-induced NF-κB activation by targeting the Cav1 protein.

### 2.8. Biolayer Interference (BLI) Assay

**Figure 7:**
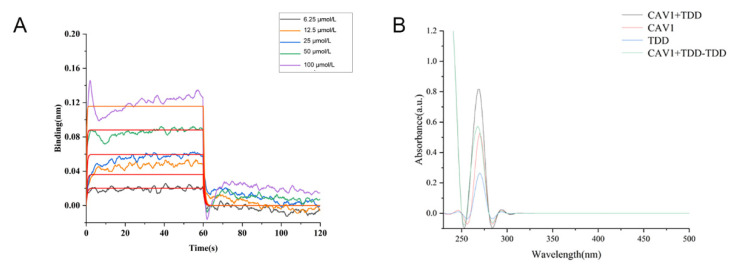
(**A**) BLI analysis of TDD binding to Cav1, (**B**) UV-vis absorption Spectra of TDD and Cav1 system.

As shown in Figure 7A, TDD bound to Cav1 in a concentration-dependent manner, with an equilibrium dissociation constant (K_D_) value of 2.90 × 10^−5^ mol/L.

### 2.9. UV-Vis Spectroscopy

A strong absorption peak was observed at 280 nm, representing the characteristic amino acid residues of the Cav1 protein (Figure 7B). In the presence of TDD, the absorption peak decreased, implying changes of aromatic acid residues. There was no overlap between the two spectrums, suggesting that a ground state complex was formed between TDD and Cav1.

### 2.10. Fluorescence Spectroscopy

**Figure 8:**
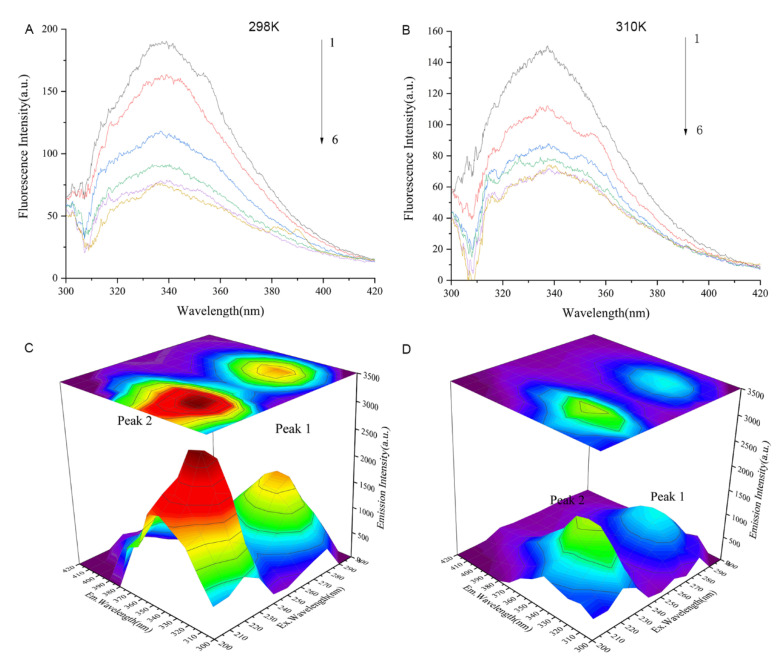
(**A**) Fluorescence quenching spectra of different concentrations of TDD on Cav1 protein at 298 K and (**B**) Fluorescence quenching spectra of different concentrations of TDD on Cav1 protein at 310 K. 1→6:0, 1.0 × 10^−7^, 2.0 × 10^−7^, 3.0 × 10^−7^, 4.0 × 10^−7^, and 5.0 × 10^−7^ mol/L. (**C**) Three-dimensional fluorescence spectra of CAV-1 and (**D**) Three-dimensional fluorescence spectra of TDD-CAV-1 system. TDD: 5 × 10^−7^ mol/L.

The fluorescence spectra of Cav1 in the absence or presence of TDD at 298 k or 310 K are represented in Figure 8A,B. The maximum emission peak of Cav1 was at 340 nm. The intensity of this peak decreased with increasing concentration of TDD.

As previously reported [35,36], the fluorescence quenching data were analyzed by the Stern–Volmer equation:

$$\frac{F_{0}}{F}=1+K_{sv}\left[Q\right]=1+K_{q}\tau_{0}\left[Q\right]$$

As shown in Table 1 and Supplementary Figure S2A, the bimolecular quenching constant (*K_q_*) between Cav1 and TDD was higher than the maximum value of the diffusion collision rate constant of the biopolymer (2 × 10^10^ M^−1^ s^−1^). The dynamic quenching constant (*K_SV_*) of the proteins showed an opposite tendency with increasing temperature, indicating that the probable quenching of Cav1 by TDD followed the static quenching mechanism.

As previously reported [37], the binding constant (*Kb*) and the number of binding sites (*n*) in the compound-protein complex were calculated by a double logarithmic curve equation.

As shown in in Table 2 and Supplementary Figure S2B, the computing binding site numbers were near to 1, which meant that there was a single binding site on Cav1 for TDD. The values of K_a_ decreased with increasing temperature, which meant that the binding ability between Cav1 and TDD was unstable with the rising temperature.

As previously reported [36], the driving force of Cav1-TDD interaction was evaluated by the van ’t Hoff equation:

△G=−RTlnK

$$\ln\frac{K_{2}}{K_{1}}=\left(\frac{1}{T_{1}}-\frac{1}{T_{2}}\right)\frac{△H}{R}$$

$$△S=\frac{△H-△G}{T}$$

The values of Δ*H*, Δ*G*, and Δ*S* obtained at different temperatures are shown in Table 3. The values of Δ*G* were negative, indicating the spontaneity between Cav1 and TDD. Regarding Δ*H*, the value was −626.55 kJ·mol^−1^, implying that the binding process was exothermic. According to a previous study [38], hydrogen bonds and van der Waals forces play a significant role in compound-protein interactions when Δ*H* < 0 and Δ*S* < 0. The negative values of Δ*H* and Δ*S* (−1.93 J·K^−1^) suggested that hydrogen bonds and van der Waals forces were involved in the interaction between Cav1 and TDD.

Further evidence of conformational changes of the Cav1 protein and the Cav1-TDD complex was provided by the three-dimensional (3D) fluorescence spectroscopy (Figure 6C,D). Peak A (λ_ex_ = 230 nm, λ_em_ = 320 nm) exhibited the fluorescence spectral behavior of polypeptide backbone structures. Peak B (λ_ex_ = 280 nm, λ_em_ = 350 nm) was identified as tryptophan and tyrosine residues. The intensity of peak A and peak B of Cav1 decreased with the addition of TDD, indicating that the interaction between TDD and Cav1 facilitates unfolding of the polypeptide chain of Cav1.

### 2.11. Molecular Docking

**Figure 9:**
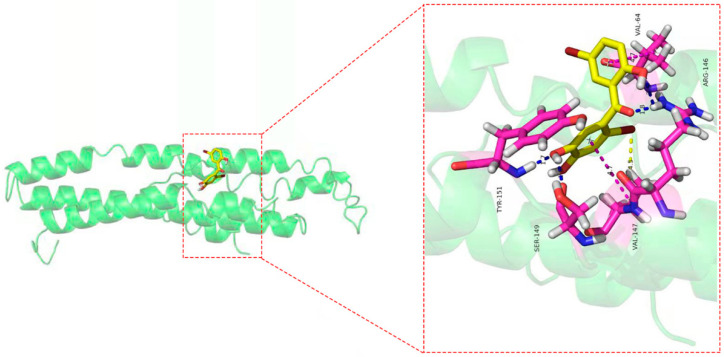
The docking results of TDD to CAV-1 protein: blue lines represent the hydrogen bond, yellow lines represent the hydrophobic effect, and blue lines represent the π-π bond in the picture.

As shown in Figure 9, eight amino acid residues took part in the binding interactions between Cav1 and TDD. These amino acid residues were Val64, Arg146, Val147, Ser149, and Tyr151. Molecular interactions between Cav1 and TDD consisted of hydrogen bonds, π-π bond and hydrophobic interactions.

## 3. Discussion

We investigated the anti-inflammatory effects of TDD in EA.hy926 cells, showing that TDD inhibited LPS-induced inflammation through the NF-κB signaling pathway. As a potential mechanism, we suggested that TDD can specifically bind to the Cav1 protein and attenuate vascular inflammation. The docking studies proposed a reasonable binding model between TDD and Cav1, promoting the Cav1 protein as a potential target to prevent vascular inflammation.

Inflammation processes accelerate the atherosclerotic process and increase the risk of cardiovascular morbidity and mortality [39,40,41]. LPS, a component of the outer membrane of Gram-negative bacteria, is a strong pro-inflammatory mediator and is extensively used in models for studying inflammation [13,14]. Although the primary human umbilical vein endothelial cells (HUVECs) are most commonly utilized in these studies [42], they have special nutrient requirements. The EA.hy926 cells maintain the biological properties of HUVECs [43,44,45,46] and are considered an in vitro model for investigation of endothelial cell functions [47,48,49,50].

It is generally known that TLR4 is one of the most important ligands of LPS on endothelial cells in atherosclerotic lesions [9]. LPS induces TLR4-mediated NF-κB activation, leading to the production of pro-inflammatory cytokines [51]. Previous studies demonstrated that TDD treatment inhibited LPS-induced inflammation in EA.hy926 cells by regulating NF-κB activation [32]. To determine whether TDD exerts its anti-inflammatory activity through TLR4-mediated signaling, we explored TLR4 expression via qRT-PCR and Western blot; however, the results did not reveal that TDD inhibited LPS-induced TLR4 activity. These results indicate that TDD inhibits LPS-induced inflammation through mechanisms other than TLR4 inhibition in EA.hy926 cells. To extend this observation, we investigated TDD’s preventive effect on LPS-induced inflammatory responses in the arterial walls of mice [52,53,54]. The study findings represent the first observation of TDD’s in vivo effect on vascular inflammation. This protective effect was associated with the suppression of inflammatory cell infiltration in vascular tissues, IL-6, and TNF-α secretion in serum, and inflammatory cytokine transcription in the arterial wall, but these effects did not involve TLR4, thereby indicating that TDD modulated the LPS-induced vascular inflammation both in vitro and in vivo without inhibiting LPS-induced TLR4 activity. Therefore, we first used the iTRAQ technique to identify potential targets and elucidate the mechanism underlying TDD’s effects on LPS-treated EA.hy926 cells.

According to the pertussis toxin-sensitive pathway, we found that TDD inactivated the NF-κB signaling pathway. The nuclear transcription factor NF-κB plays a crucial role in various biological processes, including immune response and inflammation [55]. It is inactivated through association with the sequestering inhibitory protein IκBα in the cytoplasm. NF-κB activation requires IκBα phosphorylation and subsequent degradation through ubiquitination [56]. When activated, NF-κB enters the nucleus and promotes inflammatory gene transcription. Of interest, we previously demonstrated that TDD inhibited the nuclear translocation of NF-κB in LPS-stimulated EA.hy926 cells [32], hypothesizing that TDD might suppress IκBα phosphorylation and subsequent degradation. In this study, we illustrated that TDD inhibited NF-κB activation by interfering with the phosphorylation and subsequent degradation of IκBα.

Considering the differentially expressed proteins between LPS and LPS + TDD groups, we focused on Cav1, a protein known to regulate cholesterol distribution, signal transduction, cell migration, and endocytic vesicular trafficking [57,58]. We previously suggested that TDD can attenuate LPS-induced inflammation through the regulation of cell adhesion molecules and inflammatory cytokines [32]. In the present study, we demonstrated that TDD suppressed LPS-induced activation of NF-κB due to a decreased IκBα phosphorylation and consequent degradation. When the Cav1 gene expression was knocked down, the anti-inflammatory activities of TDD were abolished.

To further explore the relationship between TDD and Cav1, we explored a potential interaction between them. TDD could specifically bind to Cav1, forming a new complex with a static quenching. The main driving forces were hydrogen bonds and van der Waals forces. TDD formed stable hydrogen bonds with Val64, Arg146, Ser149, and Tyr151, hydrophobic interaction, and π-π bond with Val147 of Cav1. Moreover, TDD facilitated unfolding of the polypeptide chain of Cav1, while the negative binding free energy of the TDD-Cav1 complex showed that the interaction was spontaneous.

**Figure 10:**
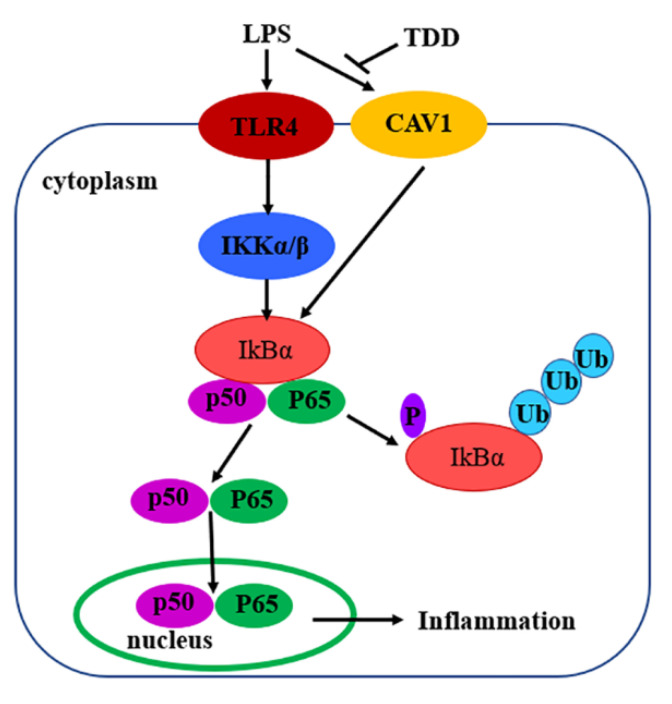
Diagram of the possible mechanisms of TDD-induced effects in LPS-stimulated EA.hy926 Cells.

In summary, the present study demonstrated that TDD inhibited LPS-induced inflammation, at least in part, via the Cav1-mediated NF-κB signaling pathway (Figure 10). TDD might be a potential anti-inflammatory compound targeting the NF-κB signaling pathway through the Cav1 protein. These findings appear to be of relevance especially for vascular inflammatory responses associated with the development of atherosclerosis. TDD may have clinical utility as a novel drug against atherosclerosis.

## 4. Materials and Methods

### 4.1. Reagents

TDD (purity, >99%) was synthesized in our laboratory as previously reported [59]. Human recombinant Cav1 were purchased from Cloud-Clone Corp. (Wuhan, China). Fetal bovine serum (FBS, SA212.02, cellmax, Beijing, China), Dulbecco’s modified Eagle’s medium (DMEM, C11965500BT, GIBCO, China), Lipopolysaccharide (LPS) and Dimethylsulfoxide (DMSO) were obtained from Sigma-Aldrich Co., Ltd. All other chemicals or solvents were of the highest grade commercially available.

### 4.2. Cell Culture

Cells derived from the human EAhy.926 EC line were obtained from the Cell Bank of the Chinese Academy of Sciences (Shanghai, China). Cells were maintained in DMEM supplemented with 10% FBS and 1% penicillin/streptomycin and in a humidified air/CO_2_ incubator (5% *v/v*) at 37 °C.

### 4.3. Animals and Treatments

BALB/c mice (male, weight = 18–22 g) were obtained from Shanxi Medical University, Taiyuan, China. Animal procedures strictly adhered to a protocol reviewed and approved by the Shanxi University of Traditional Chinese Medicine Ethics Committee and conformed to the guidelines for the Care and Use of Laboratory Animals (2018DW108).

The mice were randomly divided into five groups (six per group) as follows: control group, LPS group, and three LPS+TDD groups (4.5, 13.5, or 40.5 mg/kg TDD). TDD was administered for 5 days prior to LPS treatment (10 mg/kg, i.p.) [53]. Controls were injected with the same volume of vehicle. Animals were sacrificed 24 h after the LPS injection. The blood and aortae of mice were collected for further experiments.

### 4.4. Histopathological Evaluation

Arterial tissues were fixed in 4% paraformaldehyde, dehydrated with ethanol, and embedded in paraffin. Subsequently, the tissues were sliced into 5-μm-thick sections, stained with hematoxylin-eosin (H&E), and observed under a light microscope.

### 4.5. Enzyme-Linked Immunosorbent Assay (ELISA) 

Serum levels of TNF-α and IL-6 were measured using mouse ELISA kits according to the manufacturer’s instructions (Uscn Life Science Inc., Wuhan, China).

### 4.6. Quantitative Reverse Transcription-Polymerase Chain Reaction (qRT-PCR)

Total mRNA from EA.hy926 cells was extracted, using TRIzol reagent (9108, TaKaRa, Tokyo, Japan), followed by reverse transcription to generate cDNA according to the manufacturer’s instructions (RR047A, TaKaRa). The primer sequences used in this study are listed in Table 3. cDNA was amplified using a SYBR Premix Ex Taq™ II Kit (RR820A, TaKaRa) on the StepOnePlus™ PCR System (Applied Biosystems, Foster City, CA, USA). Raw data were calculated and normalized to the control values. GAPDH was chosen as the internal control.

### 4.7. Western Blot Analysis

The total protein of cells was prepared as previously described [32]. Sample proteins (30 μg) were separated via SDS-PAGE, transferred to nitrocellulose membranes, and blocked with 5% non-fat milk. After incubation with the indicated antibodies, the membranes were washed and incubated with the secondary antibodies. Immunoreactivity was detected using ECL reagents. The following primary antibodies were used in this study: β-actin antibody (AP0060, Bioworld, Nanjing, China), TLR4 antibody (sc-293072, Santa Cruz Biotechnology, Paso Robles, CA, USA), Cav-1 antibody (ab192869, ABCAM, Cambridge, UK), Ras homolog family member A (RhoA) antibody (2117S, Cell Signaling Technology, Danvers, MA, USA), p65 antibody (10745-1-AP, Cell Signaling Technology), p-p65 antibody (3033S, Cell Signaling Technology), IκBα antibody (4814S, Cell Signaling Technology), and p-IκBα antibody (2859S, Cell Signaling Technology).

### 4.8. Isobaric Tag for the Relative and Absolute Quantification Technique (iTRAQ)

The proteins were extracted from EA.hy926 cells using the Lysis buffer (7 mol/L Urea, 2 mol/L Thiourea, 4% CHAPS, 40 mmol/L Tris-HCl, pH 8.5) containing protease inhibitors PMSF (1 mmol/L) and EDTA (2 mmol/L). The protein concentrations were determined using BCA kits (Beyotime Institute of Biotechnology). After trypsin digestion and peptide measurement, iTRAQ labeling was performed according to the manufacturer’s protocol for 8-plex iTRAQ reagent (Applied Biosystems). The labeled samples were mixed and divided into 20 fractions, via strong cation exchange chromatography, using an LC-20AB HPLC system (Shimadzu, Kyoto, Japan). After desalting in a Strata X C18 column (Phenomenex, Torrance, CA, USA), each fraction’s supernatant was loaded on an LC-20AD nanoHPLC system (Shimadzu). Mass spectrometry (MS) using a TripleTOF 5600 System (AB SCIEX, Concord, ON, Canada) was performed after HPLC.

The Mascot search engine (Matrix Science, London, UK; version 2.3.02) was applied to analyze the MS data for protein identification. The proteins that possessed at least two unique spectra were considered for further analysis. *p* < 0.05 and fold change >1.2 denoted significance.

### 4.9. Cav1 Adenovirus Infection

EA.hy926 cells were infected with a recombinant adenovirus encoding human Cav1 (Shanghai Genechem Co., Ltd., Shanghai, China) to silence Cav1. The target Sequence of shRNAs was CGGGACGTGGTCAAGATTGACTTTCTCGAGAAAGTCAATCTTGACCACGTCTTTT. A recombinant adenovirus carrying GFP was used as the negative vector. The infection process was performed according to the manufacturer’s instructions.

### 4.10. BLI Assay

The interaction between Cav1 and TDD was determined by using BLI technology on the ForteBio Octet RED 96 system. PBS with 5% DMSO was used as working buffer throughout the trial. First, the Cav1 protein (1.0 × 10^−5^ mol/L) was incubated with biotin. TDD were diluted into different concentrations (6.25, 12.5, 25, 50, 100 mol/L). Then, the K_d_ values were calculated by the SSA sensor (Fortebio, Menlo Park, CA, USA) using ForteBio Data Analysis Software (v.11.1.0.4).

### 4.11. UV-vis Absorption Spectra

The UV-vis absorption spectra of Cav1 (1.0 × 10^−5^ mol/L) with TDD (0 and 1.0 × 10^−5^ mol/L) and TDD (1.0 × 10^−5^ mol/L) were measured in the wavelength range of 230–500 nm.

### 4.12. Fluorescence Spectrum

All fluorescence spectra of the Cav1-TDD system were collected on the F-4700 fluorescence spectrometer (Hitachi, Japan). Working voltage and scanning speed were controlled at 700 V and 240 nm/min. The Cav1 concentration was kept at 5.0 × 10^−9^ mol/L, while varying the TDD concentration (0, 1.0 × 10^−7^, 2.0 × 10^−7^, 3.0 × 10^−7^, 4.0 × 10^−7^ and 5.0 × 10^−7^ mol/L) at 298 K and 310 K. The excitation wavelength was set at 280 nm and the emission wavelength was performed from 300 nm to 420 nm. The excitation and emission slit widths were fixed at 5 nm.

The three-dimensional (3D) fluorescence spectra of Cav1 (5.0 × 10^−9^ mol/L) were recorded at 298 K in the absence and presence of TDD (5.0 × 10^−7^ mol/L) was measured on the F-4700 fluorescence spectrometer (Hitachi, Japan). The emission wavelength was performed between 300 nm and 420 nm with 5 nm interval. The excitation wavelength was recorded between 200 nm and 300 nm with 5 nm interval.

### 4.13. Molecular Docking

The molecular docking software AutoDock Vina 1.1.2 was carried out to illustrate the probable binding mode of TDD at the active site of Cav1 and find some important residues for binding. Homology modeling of Cav1 was obtained from I-TASSER (https://zhanglab.ccmb.med.umich.edu/I-TASSER/, accessed on 9 August 2022). The docking input files for the Cav1 and ligand were generated by AutoDockTools.

### 4.14. Statistical Analysis

All results are expressed as the mean ± SD. Differences between the experimental and control groups were compared using Student’s *t*-test, and *p* < 0.05 denoted statistical significance. All data analyses were performed using GraphPad Prism 5.0 (GraphPad Software Inc., San Diego, CA, USA).

## Figures and Tables

**Table 1:** *Ksv* and *Kq* values of TDD and Cav1 protein at 298 K and 310 K.

*T(K)*	*K_sv_* (L·mol^−1^) × 10^6^	*K_q_* (L·mol^−1^·s^−1^) × 10^14^	*R* ^2^
298	3.48	3.48	0.95
310	1.94	1.94	0.93

**Table 2:** *K_b_* and *n* values of different TDD and Cav1 protein at 298 K and 310 K.

*T(K)*	*K_b_*(L·mol^−1^)	*n*	*R* ^2^
298	1.09 × 10^9^	1.3907	0.96
310	6.11 × 10^4^	0.7436	0.96

**Table 3:** Sequences of forward and reverse primers for quantitative real-time Polymerase Chain Reaction.

Gene	Forward Primer Sequence (5′-3′)	Reverse Primer Sequence (5′-3′)
GAPDH (human)	TGGTGAAGACGCCAGTGGA	GCACCGTCAAGGCTGAGAAC
TLR4 (human)	TAAGGTTGCCGCTTTCACTT	TGACCGAGCAGTTTCTGAGG
MCP-1 (human)	TCAGCCAGATGCAATCAATG	AGATCTCCTTGGCCACAATG
IL-6 (human)	GTGTGAAAGCAGCAAAGAG	CTCCAAAAGACCAGTGATG
TNF-α (human)	CCTGTGAGGAGGACGAACAT	TTTGAGCCAGAAGAGGTTGAG
IL-1β (human)	TGGCAGAAAGGGAACAGAAA	CTGGCTGATGGACAGGAGAT
GAPDH (mouse)	AGGTCGGTGTGAACGGATTTG	TGTAGACCATGTAGTTGAGGTCA
TLR4 (mouse)	GAGCCGGAAGGTTATTGTGGTAGTG	TCAAGGACAATGAAGATGATGCCAGAG
MCP-1 (mouse)	CCACTCACCTGCTGCTACTCATTC	CTGCTGCTGGTGATCCTCTTGTAG
IL-6 (mouse)	GATGGATGCTACCAAATGGA	CCAGGTAGCTATGGTACTCCAGAA
TNF-α (mouse)	CCTCTAGCCCACGTCGTAGC	AGCAATGACTCCAAAGTAGACC
IL-1β (mouse)	AGCAGCACATCAACAAGAGC	AGGTGCTCATGTCCTCATCC

## Data Availability

Requests to access the datasets should be directed to the corresponding author.

## Supplementary Materials

The following supporting information can be downloaded at: https://www.mdpi.com/article/10.3390/molecules27092884/s1, Figure S1: The signaling pathways of pertussis. Figure S2: A: Interaction of TDD with Cav1 at 298 K and 310 K; B: Diagram of binding constants and binding sites between TDD and Cav1.

## Sample Availability

Samples of the compounds are not available from the authors.
