# Supplementary figures and images for: 5,2′-Dibromo-2,4′,5′-trihydroxydiphenylmethanone Inhibits LPS-Induced Vascular Inflammation by Targeting the Cav1 Protein
Source: Molecules. 2022 Apr 30;27(9):2884. doi: 10.3390/molecules27092884 (PMC9101869; doi:10.3390/molecules27092884)

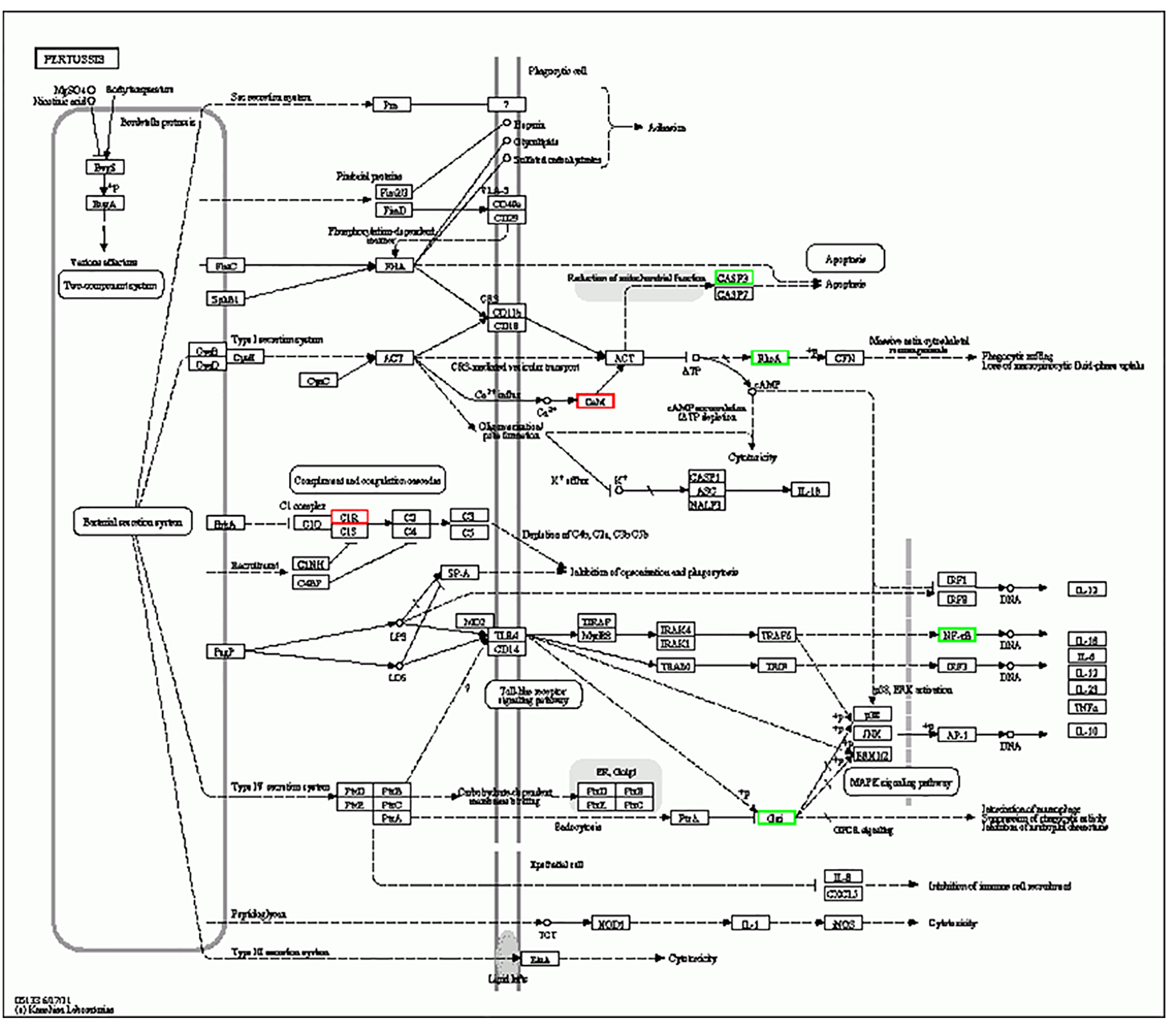

Supplement: Supplementary file 1 [file molecules-27-02884-s001.zip › Figure S1.tif]

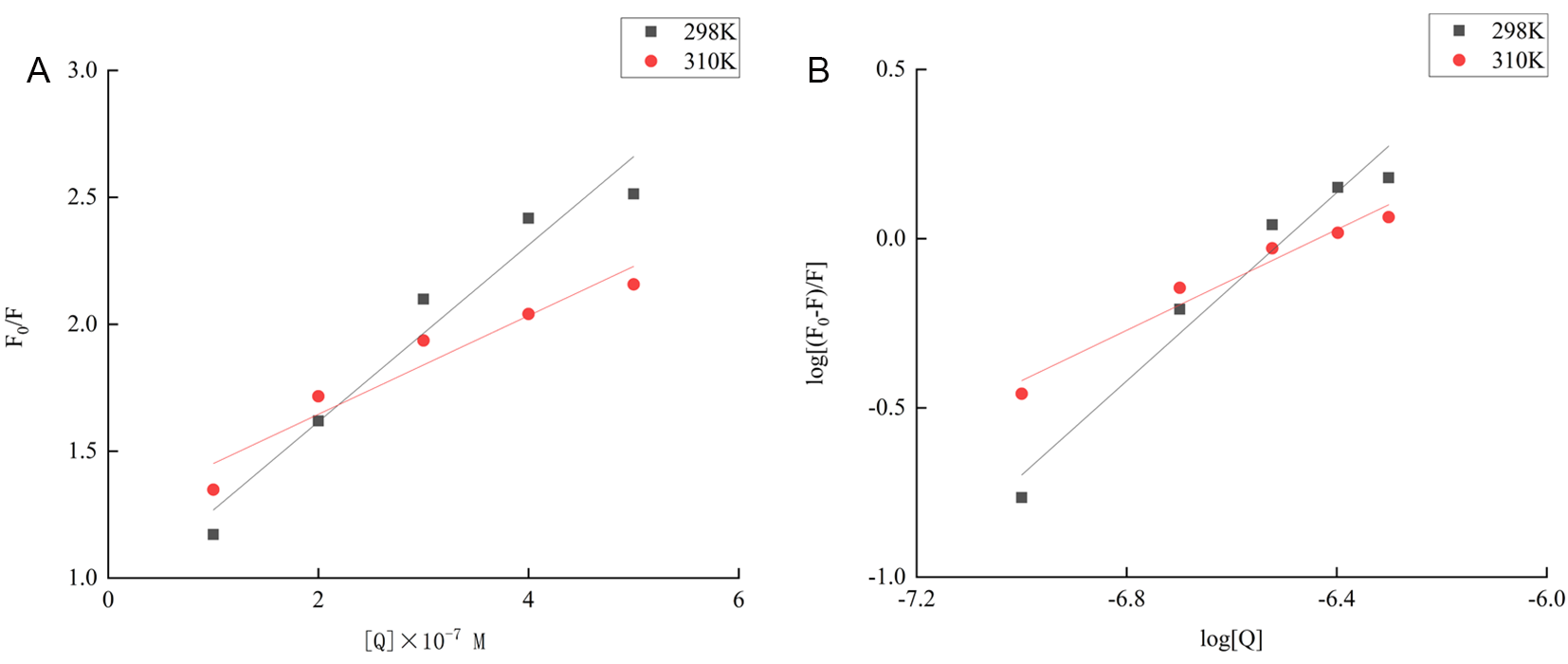

Supplement: Supplementary file 1 [file molecules-27-02884-s001.zip › Figure S2.tif]
